# More and More Coronaviruses: Human Coronavirus HKU1

**DOI:** 10.3390/v1010057

**Published:** 2009-06-11

**Authors:** Patrick C. Y. Woo, Susanna K. P. Lau, Cyril C. Y. Yip, Yi Huang, Kwok-Yung Yuen

**Affiliations:** 1 State Key Laboratory of Emerging Infectious Diseases, The University of Hong Kong, Hong Kong, China; 2 Research Centre of Infection and Immunology, The University of Hong Kong, Hong Kong, China; 3 Department of Microbiology, The University of Hong Kong, Hong Kong, China

**Keywords:** coronavirus, human, HKU1, novel

## Abstract

After human coronaviruses OC43, 229E and NL63, human coronavirus HKU1 (HCoV-HKU1) is the fourth human coronavirus discovered. HCoV-HKU1 is a group 2a coronavirus that is still not cultivable. The G + C contents of HCoV-HKU1 genomes are 32%, the lowest among all known coronaviruses with complete genome sequences available. Among all coronaviruses, HCoV-HKU1 shows the most extreme codon usage bias, attributed most importantly to severe cytosine deamination. All HCoV-HKU1 genomes contain unique tandem copies of a 30-base acidic tandem repeat of unknown function at the *N*-terminus of nsp3 inside the acidic domain upstream of papain-like protease 1. Three genotypes, A, B and C, of HCoV-HKU1 and homologous recombination among their genomes, are observed. The incidence of HCoV-HKU1 infections is the highest in winter. Similar to other human coronaviruses, HCoV-HKU1 infections have been reported globally, with a median (range) incidence of 0.9 (0 – 4.4) %. HCoV-HKU1 is associated with both upper and lower respiratory tract infections that are mostly self-limiting. The most common method for diagnosing HCoV-HKU1 infection is RT-PCR or real-time RT-PCR using RNA extracted from respiratory tract samples such as nasopharyngeal aspirates (NPA). Both the *pol* and nucleocapsid genes have been used as the targets for amplification. Monoclonal antibodies have been generated for direct antigen detection in NPA. For antibody detection, *Escherichia coli* BL21 and baculovirus-expressed recombinant nucleocapsid of HCoV-HKU1 have been used for IgG and IgM detection in sera of patients and normal individuals, using Western blot and enzyme-linked immunoassay.

## 1. Introduction

Coronaviruses are positive-sense, single-stranded RNA viruses found in humans and a wide variety of animals. In humans, coronaviruses are mainly causes of respiratory tract infections, whereas in animals, they can cause respiratory, enteric, hepatic and neurological diseases of varying severity. As in other RNA viruses, the infidelity of RNA-dependent RNA polymerase results in high mutation rates. In addition, coronaviruses possess a unique mechanism of viral replication that leads to high frequencies of recombination, as well as the largest genome size (26.4 to 31.7 kb) among all known RNA viruses that gives this family of viruses extra plasticity [18,22,43]. All these factors have allowed the coronaviruses to adapt to new hosts and ecological niches. Traditionally, coronaviruses were classified into groups 1, 2 and 3, with groups 1 and 2 consisting of mammalian coronaviruses and group 3 being avian coronaviruses [2,22,47]. In 2008, the Coronavirus Study Group of the International Committee for Taxonomy of Viruses proposed three genera, *Alphacoronavirus*, *Betacoronavirus* and *Gamma-coronavirus*, to replace these three traditional groups of coronaviruses (http://talk.ictvonline.org/cfs-filesystemfile.ashx/__key/CommunityServer.Components.PostAttachments/00.00.00.06.26/2008.085_2D00_122V.01.Coronaviridae.pdf).

The SARS epidemic that originated from southern China in 2003 has boosted interest in all areas of coronavirus research, most notably, coronavirus biodiversity and genomics. Before 2003, there were only 10 coronaviruses with complete genomes available, including only two human coronaviruses, human coronavirus 229E (HCoV-229E) and human coronavirus OC43 (HCoV-OC43). These two human coronaviruses were discovered in the 1960s, with HCoV-229E being a group 1 coronavirus and HCoV-OC43 a group 2 coronavirus [17, 34]. After the SARS epidemic, up to December 2008, 16 novel coronaviruses were discovered and their complete genomes sequenced. Among these 16 previously unrecognized coronaviruses were two more human coronaviruses, human coronavirus NL63 (HCoV-NL63) and human coronavirus HKU1 (HCoV-HKU1) [37,39], ten other mammalian coronaviruses and four avian coronaviruses [7,16,23,24,27,28,33,40,41,44,46]. HCoV-NL63 is a group 1 coronavirus whereas HCoV-HKU1 is a group 2 coronavirus. In just a few years after their discoveries, numerous reports throughout the world had described the presence of HCoV-NL63 and HCoV-HKU1 in patients with respiratory infections in their corresponding countries. In this article, we reviewed our current understanding of the classification, virology, epidemiology, clinical diseases, laboratory diagnosis, treatment and prevention of HCoV-HKU1.

## 2. Classification and virology

**Figure 1.**
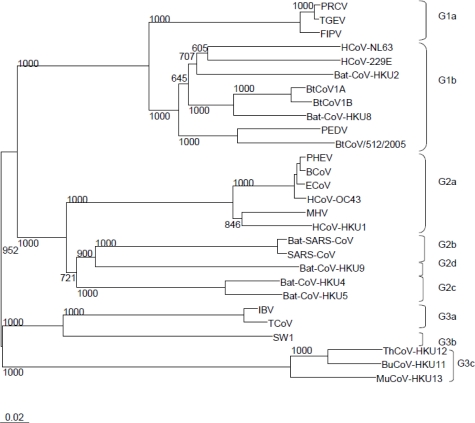
Phylogenetic analysis of RNA-dependent RNA polymerases of coronaviruses with complete genome sequences available by the end of 2008. The tree was constructed by neighbor joining method using Kimura's two-parameter correction and bootstrap values calculated from 1000 trees. Nine hundred and fifty eight amino acid positions were included in the analysis. The scale bar indicates the estimated number of substitutions per 50 amino acids. HCoV-229E, human coronavirus 229E (NC_002645); PEDV, porcine epidemic diarrhea virus (NC_003436); TGEV, porcine transmissible gastroenteritis virus (NC_002306); FCoV, feline coronavirus (AY994055); PRCV, porcine respiratory coronavirus (DQ811787); HCoV-NL63, human coronavirus NL63 (NC_005831); bat-CoV-HKU2 (EF203064), HKU4 (NC_009019), HKU5 (NC_009020), HKU8 (NC_010438), HKU9 (NC_009021), 1A (NC_010437), 1B (NC_010436), 512/2005 (NC_009657); HCoV-HKU1, human coronavirus HKU1 (NC_006577), HCoV-OC43, human coronavirus OC43 (NC_005147); MHV, mouse hepatitis virus (NC_006852); BCoV, bovine coronavirus (NC_003045); PHEV, porcine hemagglutinating encephalomyelitis virus (NC_007732); ECoV, equine coronavirus (NC_010327); SARS-CoV, SARS coronavirus (NC_004718); bat-SARS-CoV-HKU3, bat-SARS coronavirus HKU3 (NC_009694); IBV, infectious bronchitis virus (NC_001451); TCoV, turkey coronavirus (NC_010800); SW1, beluga whale coronavirus (NC_010646); BuCoV-HKU11, Bulbul coronavirus HKU11 (NC_011548); ThCoV-HKU12, Thrush coronavirus HKU12 (NC_011549); MuCoV-HKU13, Munia coronavirus HKU13 (NC_011550).

In the current system of classification in which group 2 coronaviruses are classified into group 2a, group 2b and the recently proposed groups 2c and 2d, HCoV-HKU1 is a group 2a coronavirus. Phylogenetically, no close relative with less than 10% nucleotide difference in any of the genes is present (Figure 1)

**Figure 2.**
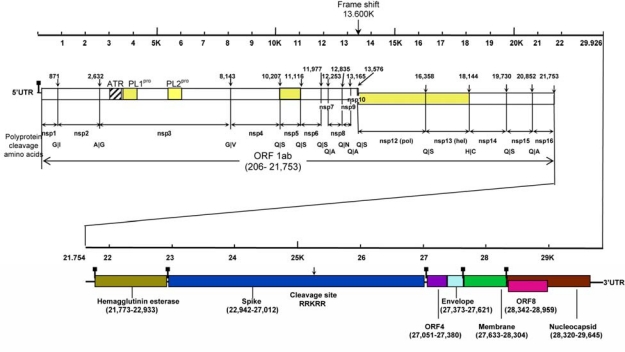
Genome organization of HCoV-HKU1. Predicted ORF 1ab, encoding the nonstructural polyproteins (nsp1 to nsp16) and ORFs encoding the HE, S, E, M and N structural proteins are indicated. Arrows indicate putative cleavage sites (with the corresponding nucleotide positions) of the replicase polyprotein encoded by ORF 1ab (amino acids at boundaries also shown) and S protein. Putative positions of TRS are marked by▪. Major parts of ORF 1ab (PL1^pro^, PL2^pro^, 3CL^pro^, *pol* and helicase) encoding key enzymatic activities are colored in yellow. ATR represents the acidic tandem repeat in nsp3.

At the moment, 22 HCoV-HKU1 genomes have been sequenced [43]. The sizes of the genomes of HCoV-HKU1 range from 29,295 to 30,097 nucleotides. The G + C contents of HCoV-HKU1 genomes are 32%, the lowest among all known coronaviruses with complete genome sequences available. The genome organization of HCoV-HKU1 is similar to that of other coronaviruses, with the characteristic gene order 5′-replicase ORF1ab, spike (S), envelope (E), membrane (M), nucleocapsid (N)-3′ (Figure 2). Additional genomic features of HCoV-HKU1 that are similar to all other group 2a coronaviruses include a putative transcription regulatory sequence (TRS) of CUAAAC, two papain-like protease domains (PL1^pro^ and PL2^pro^) in nsp3 of ORF1ab, a haemagglutinin esterase (HE) gene between ORF1ab and S, and an internal ribosomal entry site upstream to the initiation codon of E [39]. Among all coronaviruses with complete genomes available, HCoV-HKU1 shows the most extreme codon usage bias, attributed most importantly to severe cytosine deamination that has led to C → U changes [45].

The ORF1ab replicase polyprotein is putatively cleaved by its papain-like proteases and 3C-like protease (3CL^pro^) into 16 nonstructural proteins (nsp1 to nsp16) homologous to the corresponding ones in other coronaviruses. Analysis of the putative cleavage sites of the 3CL^pro^ revealed a unique putative cleavage site at the junction between nsp13 and nsp14, which was also recognized in HCoV-NL63 [38]. In addition to this unique putative cleavage site, all HCoV-HKU1 genomes contain tandem copies of a 30-base acidic tandem repeat (ATR) (variable numbers of perfect repeats of NDDEDVVTGD in the amino acid sequence followed by variable numbers of imperfect repeats) at the N-terminus of nsp3 inside the acidic domain upstream of PL1^pro^ [43]. This phenomenon was also observed in the two strains of HCoV-HKU1 found in France with this region of the genome characterized [35]. Although the function of the ATR is not known, these tandem copies of ATR have made the nsp3 of HCoV-HKU1 the longest nsp3 among all coronaviruses with complete genomes available.

At least three genotypes, genotype A, B and C, of HCoV-HKU1, with inter-genotypic homologous recombination, have been observed. When the *pol*, S and N genes of clinical strains HCoV-HKU1 were analyzed, it was observed that the strains fell into two clusters, designated genotypes A and B [25,42]. Interestingly, there were a few strains in which the sequences of *pol* genes were clustered with genotype A but those of S and N genes were clustered with genotype B [25,42]. Subsequent sequencing and analysis of 22 complete HCoV-HKU1 genomes found that there were three genotypes of HCoV-HKU1 and significant numbers of homologous recombination events have occurred among the three genotypes [43]. The most notable example was in a stretch of 29 bases at the 3′ end of nsp16, in which recombination between genotype A and genotype B has led to the generation of genotype C [43]. This represented the first example of homologous recombination in human coronavirus, and was also the first study to describe a distribution of natural recombination spots in the entire genome of field isolates of a coronavirus [43]. Although no complete genome sequence is available for HCoV-HKU1 strains found outside Hong Kong, sequences of fragments of *pol*, S and N of the strains suggested that all three genotypes are probably distributed globally. Analysis of a single gene is not sufficient for genotyping of HCoV-HKU1, but would require sequencing of at least two gene loci, one from nsp10 to nsp16, such as *pol* or helicase, and another from HE to N, such as S or N.

Although HCoV-HKU1 is still not cultivable using a wide variety of cell lines, neuron-glia culture and intracerebral inoculation of suckling mice, the biogenesis, subcellular localization and intracellular trafficking of S in HCoV-HKU1 as well as its interaction with major histocompatibility complex class I C molecule (HLA-C) were characterized recently [4, 6]. Results of these studies confirmed that S of HCoV-HKU1 was *N*-glycosylated in the endoplasmic reticulum (ER) with high-mannose *N*-glycans, and it was first distributed in the ER and Golgi, subsequently also detected in vesicles throughout the cytoplasm, and finally on the cell surface. Cleavage of S into S1 (Endo H deglycosylation resistant) and S2 (Endo H deglycosylation sensitive) that was inhibited by the furin or furin-like enzyme inhibitor, peptidyl chloromethylketone was also observed, confirming the bioinformatics prediction of the presence of cleavage site between S1 and S2 [6]. Using HCoV-HKU1 S pseudotyped virus, human alveolar epithelial A549 cells were shown to be the most susceptible cell line [4]. Using an A549 cDNA expression library transduced into the non-permissive, baby hamster kidney cell line BHK-21 for detecting proteins that bind HCoV-HKU1 S_1-600_ glycoprotein, independent clones with inserts encoding HLA-C were fished out. Further experiments also suggested that HLA-C is involved in the attachment of HCoV-HKU1 to A549 cells and is a potential candidate to facilitate cell entry [4].

## 3. Epidemiology

In our studies on animal coronaviruses, no HCoV-HKU1 was detected in screening more than 10,000 animal specimens in a variety of mammalian and avian species [23, 40, 41, 44]. The dn/ds ratios of all ORFs in the genomes of HCoV-HKU1 are low. Therefore, HCoV-HKU1 is stably evolving in humans, probably the only known reservoir. Similar to other respiratory viruses, HCoV-HKU1 is presumably transmitted through exchange of respiratory secretions. The incidence of HCoV-HKU1 infections is the highest in winter. Similar to other human coronaviruses, HCoV-HKU1 infections have been reported globally (Table 1) [1,3,8,9,11–15,20,21,25,29,30,32,35,36,42]. From the studies with the incidence of all four human coronaviruses examined (Table 1), the median (range) incidence of HCoV-HKU1 was 0.9 (0 – 4.4) %, with no significant difference to those of HCoV-OC43 [1.9 (0 – 6.3) %], HCoV-229E [0.4 (0 – 6.9) %] and HCoV-NL63 [1.1 (0 – 8.0) %]. Moreover, different human coronaviruses were more prevalent than the others in different studies.

The seroprevalence of HCoV-HKU1 antibody varied widely in different studies that used different antigens and methodologies for antibody detection. In a recent study on seroepidemiology of HCoV-HKU1 using *Escherichia coli* BL21 expressed recombinant S-based enzyme-linked immunosorbent assay (ELISA) and line immunoassay, it was observed that the seroprevalence of HCoV-HKU1 antibody increased from 0% in patients who were <10 years old to a plateau of 22% in patients who were 31 to 40 years old [5]. In another study on seroepidemiology of the four human coronaviruses in Germany using *E. coli* BL21-expressed recombinant N-based line immunoassay, it was noted that the seropositivity for HCoV-HKU1 in 25 healthy blood donors was 48%, similar to those for HCoV-OC43 (52%), HCoV-229E (56%) and HCoV-NL63 (60%) [26]. On the other hand, in another study of a US metropolitan population using baculovirus-expressed recombinant N-based ELISA, it was observed that the proportion of HCoV-HKU1 seropositive adults was 59.2%, significantly lower than those for HCoV-OC43 (90.8%), HCoV-229E (91.3%) and HCoV-NL63 (91.8%) [31]. Moreover, it was also observed that significantly different seropositivity rates for the various human coronaviruses were observed in individuals of different races, smoking status and socioeconomic status [31]. Further studies have to be performed to delineate whether these demographic factors confer differential risks of susceptibility to different human coronaviruses.

## 4. Clinical diseases

Similar to other human coronaviruses, HCoV-HKU1 is associated with both upper and lower respiratory tract infections. Respiratory tract infections associated with HCoV-HKU1 are indistinguishable from those associated with other respiratory viruses. For upper respiratory tract infections, most patients present with fever, running nose and cough; while for lower respiratory tract infections, fever, productive cough and dyspnea are common presenting symptoms. Most HCoVHKU1 infections are self-limiting, with only two deaths reported in patients with HCoV-HKU1 pneumonia [42]. Both had underlying diabetes mellitus, cardiovascular diseases (myocardial infarction in one and cerebrovascular accident in the other) and cancers (gastric lymphoma in one and prostatic carcinoma in the other), lymphopenia and airspace shadows in both lungs [42]. Interestingly, a recent study from rural Thailand that involved control patients showed the presence of human coronaviruses in >2% of control patients, which raised questions about the role of human coronaviruses in pneumonia [9]. At the moment, no antiviral drugs or vaccines for HCoV-HKU1 and the other human coronaviruses are available. Symptomatic and supportive treatment is the mainstay of therapy given to patients suffering from infections caused by these viruses.

In addition to respiratory tract infections, HCoV-HKU1 has been found in other illnesses. In our one-year prospective study, it was observed that HCoV-HKU1 infection was associated with febrile seizures [25]. On the other hand, in another French study, although six (17.6%) of the 34 HCoV-HKU1 infected children were admitted for epileptic seizures, HCoV-HKU1 infections were not shown to be associated with febrile seizures [36]. In one study, HCoV-HKU1 was detected in the stool samples of two patients with respiratory tract infections but no gastrointestinal tract symptoms, but it was not detected in patients with diarrhea [35]. In another study, HCoV-HKU1 was detected in a liver transplant recipient with hepatitis, which other causes of hepatitis, such as graft rejection, cytomegalovirus, etc. were excluded [11]. The significance of HCoV-HKU1 febrile seizures, gastroenteritis and hepatitis remains to be determined.

## 5. Laboratory diagnosis

The most common method for making a diagnosis of HCoV-HKU1 infection is RT-PCR or real-time RT-PCR using RNA extracted from respiratory tract samples such as nasopharyngeal aspirates (NPA). Both the *pol* and N genes have been used as the targets for amplification (Table 2). In our index patient, the viral loads in the NPA of the patient were 10^6^ to 10^7^ and 10^5^ copies/ml of NPA in the first and second weeks of illness respectively, and became undetectable from the third week of illness onwards [39]. Recently, DNA microarrays have also been used for detection of HCoV-HKU1 and other human coronaviruses, and the sensitivity was found to be similar to that of individual real-time RT-PCR [10]. In addition to nucleic acid detection, monoclonal antibodies have been generated for direct antigen detection in NPA, although the targets for the binding of the monoclonal antibodies are still not known [15].

For antibody detection, *E. coli* BL21- and baculovirus-expressed recombinant N of HCoV-HKU1 has been used for IgG and IgM detection in sera of patients and normal individuals, using Western blot and ELISA [5,26,31,39,42]. In our index patient, his serum IgG titer rose from <1:1,000 in the first week of his illness to 1:2,000 and 1:8,000 in the second and fourth weeks of his illness respectively, whereas the IgM titers in the first, second and fourth weeks were 1:20, 1:40, and 1:80 respectively [39]. Furthermore, in our retrospective study of patients with community acquired pneumonia, all the six patients with serum samples available showed four-fold changes in IgG titer and/or presence of IgM against HCoV-HKU1 [42]. Although HCoV-HKU1 is still not cultivable, a neutralization antibody test was also developed recently using HCoV-HKU1 pseudotyped virus [5].

## 6. Concluding remarks

The discovery of SARS coronavirus marked the beginning of the race of coronavirus hunting in humans and animals. In just a few years, the number of “coronavirus” papers found by Medline search has doubled and the number of coronaviruses with complete genomes available has tripled. With this increase in the number of coronaviruses and genomes, we are starting to appreciate the diversity of coronaviruses. Moreover, comprehensive and user-friendly databases for efficient sequence retrieval and the ever-improving bioinformatics tools have further enabled us to improve our understanding of the phylogeny and genomics of coronaviruses [19]. With the increasing number of coronaviruses, more and more closely related coronaviruses from distantly related animals have been observed. The most notable example related to human coronaviruses is the clustering of HCoV-OC43, bovine coronavirus and porcine hemagglutinating encephalomyelitis virus. With more and more coronaviruses discovered, we will be able to understand the origin of the various human coronaviruses, and more importantly, the secret behind their mechanisms of interspecies transmission.

## Figures and Tables

**Table 1.** Clinical studies on HCoV-HKU1

**Studies**	**Ref.**	**Place of study**	**No. of patients/specimens**	**Patient characteristics**	**Duration of study**	**No. (%) of patients/specimens positive for HCoV-HKU1**	**No. (%) of HCoV-HKU1 positive patients with underlying diseases**	**No. (%) of patients/specimens positive for other human coronaviruses**			**Outcome**
								**HCoV-OC43**	**HCoV-229E**	**HCoV-NL63**	
1	[42]	Hong Kong	418 patients	Hospitalized patients with community-acquired pneumonia	12 months (Mar 2003 – Mar 2004)	10 (2.4)	8 (80)	Not performed	Not performed	Not performed	2 died

2	[32]	Australia	324 specimens	Children presented to Queensland hospitals or general practitioners with acute respiratory tract infections	4 months (May 2004 – Aug 2004)	10 (3.1)	Not mentioned	11 (3.4)	1 (0.3)	0 (0)	Not mentioned

3	[35]	France	135 patients	Hospitalized patients with respiratory symptoms	2 months (Feb 2005 – Mar 2005)	6 (4.4)	3 (50)	2 (1.5)	0 (0)	2 (1.5)	Not mentioned

4	[11]	USA	1048 respiratory specimens from 851 children	Specimens from emergency department, inpatient wards, intensive care units, and hospital-affiliated primary care outpatient clinic	12 months (Dec 2001 – Dec 2002)	9 (1) patients	5 (56)	Not performed	Not performed	Not performed	Not mentioned

5	[25]	Hong Kong	4181 specimens	Hospitalized patients with acute respiratory tract infections	12 months (April 2004 – Mar 2005)	13 (0.3)	8 (62)	53 (1.3)	4 (0.1)	17 (0.4)	All survived

6	[14]	Switzerland	540 specimens from 279 adults	Hospitalized patients with respiratory disease	20 months	4 (1.4) patients	Not mentioned	12 (4.3) patients	7 (2.5) patients	6 (2.2) patients	Not mentioned

7	[12]	Italy	2060 children	Children who attended the emergency department with an acute disease excluding trauma	5 months (Nov 2003 – Mar 2004)	0 (0)	-	17 (0.8)	42 (2)	20 (1)	-

8	[29]	Italy	227 children	Hospitalized children with acute respiratory infection or related conditions at the Pediatric Department	12 months (Oct 2004 – Sep 2005)	0 (0)	-	6 (2.6)	0 (0)	1 (0.4)	-

9	[15]	Italy	685 specimens from 426 patients	Hospitalized patients with acute respiratory tract infections	7 months (Nov 2005 – May 2006)	10 (2.3) patients	8 (80)	8 (1.9) patients	20 (4.7) patients	11 (2.6) patients	Not mentioned

10	[8]	Korea	231 children	Hospitalized children with acute expiratory wheezing	10 months (Feb 2006 – Nov 2006)	0 (0)	-	3 (1.3)	2 (0.9)	3 (1.3)	-

11	[9]	Thailand	734 patients	Patients hospitalized with pneumonia	Study year 1: Sep 2003 – Aug 2004	3 (0.4)	Not mentioned	31 (4.2)	3 (0.4)	7 (1) 1	Not mentioned
			1156 patients	Patients hospitalized with pneumonia	Study year 2: Sep 2004 – Oct 2005	9 (0.8)		4 (0.4)	7 (0.6)	(0.1)	
			513 patients	Outpatients with influenza-like illness	Study year 2: Sep 2004 – Oct 2005	1 (0.2)		0 (0)	2 (0.4)	9 (1.8)	

12	[21]	USA	1043 children	Outpatients, emergency department patients and inpatients with acute respiratory illness	12 months (Oct 2003 – Sep 2004)	28 (2.7)	Not mentioned	19 (1.8)	8 (0.8)	11 (1.1)	Not mentioned

13	[30]	Switzerland	112 infants	Infants followed prospectively during their first year of life for any respiratory or other disease symptoms and their treatment by weekly telephone interviews	69 months (Apr 1999 – Dec 2004)	1 (0.9)	Not mentioned	7 (6.3)	3 (2.7)	9 (8)	Not mentioned

14	[36]	France	1002 specimens from 928 children	Hospitalized children with respiratory or general symptoms	9 months (Sep 2004 – May 2005)	38 (3.8) specimens	8 (24) of 34 patients	27 (2.9) specimens	2 (0.2) specimens	33 (3.6) specimens	All survived

15	[20]	Jordan	325 children	Children with acute respiratory infection admitted to the pediatric wards	6 months (Dec 2003 – May 2004)	0 (0)	-	Not performed	Not performed	4 (1.2)	-

16	[3]	Italy	322 infants	Hospitalized infants with acute respiratory disease	24 months (Oct 2004 – Sep 2006)	6 (1.9)	Not mentioned	11 (3.4)	1 (0.3)	10 (3.1)	Not mentioned

17	[1]	Italy	85 infants	Infants hospitalized for the first acute episode of wheezing	6 months (Oct 2005 – Mar 2006)	1 (1.2)	Not mentioned	2 (2.4)	0 (0)	0 (0)	Not mentioned

18	[13]	France	159 specimens	Specimens collected from mother-child couples admitted in labor to the Gynecology-Obstetrics Unit	18 months (Jul 2003 – Jun 2004 and Mar 2005 – Aug 2005)	1 (0.6)	Not mentioned	0 (0)	11 (6.9)	0 (0)	Not mentioned

**Table 2.** Gene targets and primer sequences for HCoV-HKU1 detection

Studies	References	Place of study	Detection methods	Gene target	Target band size	Primer sequences
1	[42]	Hong Kong	RT-PCR	*pol*	453 bp	Forward 5′-AAAGGATGTTGACAACCCTGTT-3′ Reverse 5′-ATCATCATACTAAAATGCTTACA-3′
2	[32]	Australia	RT-PCR	*pol*	453 bp	Forward 5′-AAAGGATGTTGACAACCCTGTT-3′ Reverse 5′-ATCATCATACTAAAATGCTTACA-3′
3	[35]	France	RT-PCR	N	443 bp	Forward 5′-ACCAATCTGAGCGAAATTACCAAAC-3′ Reverse 5′-CGGAAACCTAGTAGGGATAGCTT-3′
4	[11]	USA	RT-PCR	*pol*	440 bp	Forward 5′-GGTTGGGATTATCCTAAATGTGA-3′ Reverse 5′-CCATCATCACTCAAAATCATCATA-3′
5	[25]	Hong Kong	RT-PCR	*pol*	453 bp	Forward 5′-AAAGGATGTTGACAACCCTGTT-3′ Reverse 5′-ATCATCATACTAAAATGCTTACA-3′
6	[14]	Switzerland	Real-time RT-PCR	*pol*	506 bp	Forward 5′-GAATTTTGTTGTTCACATGGTGATAGA-3′ Reverse 5′-GCAACCGCCACACATAACTATTT-3′ Probe 5′-FAM-TTTATCGCCTTGCGAATGAATGTGCTC-TAMRA-3′
7	[12]	Italy	Real-time RT-PCR	N	64 bp	Forward 5′-AGTTCCCATTGCTTTCGGAGTA-3′ Reverse 5′-CCGGCTGTGTCTATACCAATATCC-3′ Probe 5′-FAM-CCCCTTCTGAAGCAA-MGB-3′
8	[29]	Italy	RT-PCR	*pol*	440 bp	Forward 5′-GGTTGGGACTATCCTAAGTGTGA-3′ Reverse 5′-CCATCATCAGATAGAATCATCATA-3′
9	[15]	Italy	RT-PCR	N	516 bp	Forward 5′-CAGTGTTTTGGTAAAAGAGGACC-3′ Reverse 5′-TACCACCTAGTGTCGAATTAGG-3′
				*pol*	250 bp	Forward 5′-ACTCAAATGAATTTAAAATATGC-3′ Reverse 5′-TCACATTTAGGATAATCCCA-3′
10	[8]	Korea	RT-PCR	*pol*	921 bp	Forward 5′-GTTCAAGTGTCGCTGTTCA-3′ Reverse 5′-CTATCATTATCACAATCCACAG-3′
				*pol*	989 bp	Forward 5′-GGGTATGAAGTATCATCCTA-3′ Reverse 5′-GATAATCCCAACCCATAAGAAC-3′
				*pol*	921 bp	Forward 5′-CATCTTATAAAGGATGTTGAC-3′ Reverse 5′-ACAAACAACACATGCACCTACAC-3′
11	[9]	Thailand	Real-time RT-PCR	*pol*	95 bp	Forward 5′-CCTTGCGAATGAATGTGCT-3′ Reverse 5′-TTGCATCACCACTGCTAGTACCAC-3′ Probe 5′-FAM-TGTGTGGCGGTTGCTATTATGTTAAGCCTG Black Hole Quencher–1-3′
12	[21]	USA	Real-time RT-PCR	*pol*	96 bp	Forward 5′-TGGTGGCTGGGACGATATGT-3′ Reverse 5′-GGCATAGCACGATCACACTTAGG-3′ Probe 5′-6FAM-ATAATCCCAACCCATRAG-Quencher -3′
13	[30]	Switzerland	Real-time RT-PCR	*pol*	506 bp	Forward 5′-GAATTTTGTTGTTCACATGGTGATAGA-3′ Reverse 5′-GCAACCGCCACACATAACTATTT-3′ Probe 5′-FAM-TTTATCGCCTTGCGAATGAATGTGCTC-TAMRA-3′
14	[36]	France	RT-PCR	N	439 bp	Forward 5′-ATCTGARCGAAAYYAYCAAAC-3′ Reverse 5′-CGYAAACCTAGTAGGGATAGCTT-3′
15	[20]	Jordan	RT-PCR	*pol*	453 bp	Forward 5′-AAAGGATGTTGACAACCCTGTT-3′ Reverse 5′-ATCATCATACTAAAATGCTTACA-3′
16	[3]	Italy	RT-PCR	*pol*	220bp	Forward 5′-TTATGGGTTGGGATTATCCYAARTGTGAT-3′ Reverse 5′-GTACTAGCRTCACCAGAAGTYGTACCACC-3′
				*pol*	214bp	Forward2 5′-ATGGGATGGGACTATCCTAAGTGTGATAGAG-3′ Reverse2 5′-TTGCATCACCACTRCTAGTRCCACCAGGC-3′
17	[1]	Italy	RT-PCR	*pol*	440 bp	Forward 5′-GGTTGGGACTATCCTAAGTGTGA-3′ Reverse 5′-CCATCATCAGATAGAATCATCATA-3′
18	[13]	France	Real-time RT-PCR	*pol*	96 bp	Forward 5′-TGGTGGCTGGGACGATATGT-3′ Reverse 5′-GGCATAGCACGATCACACTTAGG-3′ Probe 5′-6FAM-ATAATCCCAACCCATRAG-Quencher -3′
